# Predicting Postoperative Motor Function After Brain Tumor Resection With Motor Evoked Potential Monitoring Using Decision Tree Analysis

**DOI:** 10.7759/cureus.74155

**Published:** 2024-11-21

**Authors:** Takeo Yuno, Yusuke Nakade, Mitsutoshi Nakada, Masashi Kinoshita, Masako Nakata, Shiori Nakagawa, Hiroyasu Oe, Mika Mori, Takashi Wada, Hajime Kanamori

**Affiliations:** 1 Department of Clinical Laboratory, Kanazawa University Hospital, Kanazawa, JPN; 2 Department of Neurosurgery, Kanazawa University, Kanazawa, JPN

**Keywords:** brain tumor, decision tree, machine learning, motor evoked potentials, paralysis

## Abstract

Background

Motor evoked potential (MEP) monitoring is a commonly employed method in neurosurgery to prevent postoperative motor dysfunction. However, it has low prediction accuracy for postoperative paralysis. This study aimed to develop a decision tree (DT) model for predicting postoperative motor function using MEP monitoring data.

Methodology

In this retrospective cohort study, we used datasets, comprising 14 variables including MEP amplitudes, obtained from 125 patients who underwent brain tumor resection with intraoperative MEP monitoring at our hospital. Prediction models were developed using DT and receiver operating characteristic (ROC) curve analyses. Model performance was assessed for accuracy, sensitivity, specificity, kappa (*κ*) coefficient, and area under the ROC curve (AUC) for internal and external validation. For the external validation of the classification model, we retrospectively collected data from an additional 28 patients who underwent brain tumor surgery with MEP monitoring.

Results

The amplitude of the last measured MEP and amplitude ratio were independent predictors of outcomes. The DT model achieved an accuracy of 0.921, sensitivity of 0.917, specificity of 0.923, and AUC of 0.931 using the internal test. In comparison, the ROC curve based on the amplitude of the last measured MEP achieved a sensitivity of 0.875, specificity of 0.906, and AUC of 0.941. External validation was performed and the DT model was superior to prediction by cutoff values from ROC curves in terms of accuracy, sensitivity, specificity, and* κ *coefficient.

Conclusions

Our study suggested the usefulness of DT modeling for predicting postoperative paralysis. However, this study has several limitations, such as the retrospective design and small sample size of the validation dataset. Nonetheless, the DT modeling presented in this study might be applicable to surgeries using MEP monitoring and is expected to contribute to devising treatment strategies by predicting postoperative motor function in various patients.

## Introduction

Multidisciplinary treatment improves tumor removal and survival rates in cases requiring brain tumor resection [1]. Similarly, intraoperative neurophysiological monitoring improves functional prognosis [1,2]. However, radical brain tumor resection increases the risk of poor functional prognosis [3]. New development of postoperative defects is associated with a reduction in overall survival [4]. Thus, accurate prediction of postoperative motor function in patients with brain tumors would be useful for treatment planning and improving postoperative quality of life. Intraoperative assessment of motor function is important for avoiding persistent postoperative motor dysfunction. Intraoperative motor evoked potentials (MEPs) are measured for assessing motor function during surgery [5]. Myogenic MEP monitoring detects the derivative signal of compound muscle action potentials by transcranial or direct electrical stimulation of the motor cortex [6]. However, MEPs could be affected by various factors, including anesthetics, blood pressure, and the patient’s motor function, which can lead to false positives termed anesthetic fade [7,8]. Furthermore, despite various studies evaluating MEP monitoring [8,9], whether it could help predict or prevent postoperative motor dysfunction remains unclear.

Such uncertainty about MEP monitoring may have a variety of effects, including confounding the surgeon and excessive therapeutic intervention in patients. In addition, MEP monitoring has limitations due to the lack of consensus on alert criteria for intraoperative MEP assessment and on the importance of MEP amplitude attenuation for distinguishing between temporary and permanent deficits [10]. Accurate assessment of postoperative motor function using MEP monitoring data not only solves these problems but also provides other benefits, such as enabling appropriate rehabilitation planning.

Machine learning (ML) prediction models are useful for predicting disease and postoperative outcomes [11-13]. ML has been used for preoperatively predicting postoperative motor dysfunction and other problems after adult spinal deformity surgery [14]. Among the ML algorithms used, a decision tree (DT) analysis model is simple to understand and can be easily visualized [13,15]. DT is an ML algorithm that is useful for risk and decision analysis. To date, there have been no reports on the application and usefulness of ML techniques, particularly the DT model, for predicting postoperative motor dysfunction in patients with brain tumors based on MEP monitoring data.

Therefore, in this retrospective observational study, we developed and evaluated a DT model using MEP monitoring data for postoperatively predicting motor function, over two months, based on manual muscle testing in patients who underwent brain tumor surgery.

## Materials and methods

Patients

**Figure 1:**
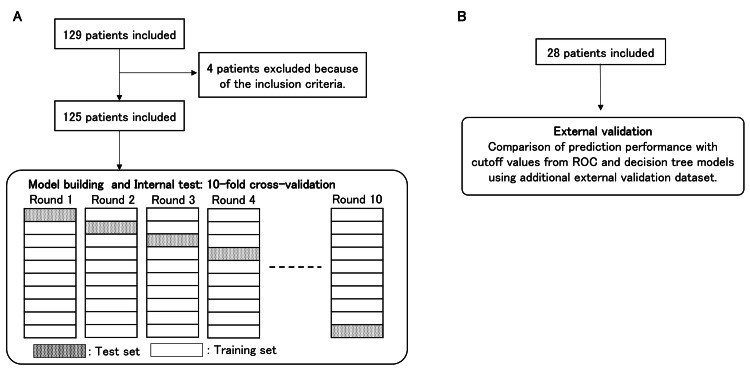
Study flow diagram. A. Decision tree model building and internal test. B. External validation. ROC: receiver operating characteristic

This retrospective observational study used routinely collected data from 129 patients who underwent brain tumor surgery with MEP monitoring at the Department of Neurosurgery, Kanazawa University Hospital, between April 2016 and February 2021. We excluded patients with undetectable baseline MEPs and those with postoperative stroke or cerebral hemorrhage that resulted in paralysis (Figure 1). Motor function was assessed in all patients at least two months after surgery. The median time for assessing motor function was 3.5 months after surgery. In the stable group, there was no postoperative motor dysfunction in the muscles that were not included in the analysis. Postoperative motor dysfunction was defined as a postoperative reduction of at least one manual muscle testing grade. Recorded patient data included baseline values, such as age, sex, body mass index, preoperative motor weakness, lesion side, tumor location, method of MEP stimulation, and MEP monitoring. We also recorded postoperative values, such as pathological diagnosis, operative time, baseline MEP amplitude, changes in intraoperative MEP response (vs. baseline amplitude), the amplitude of the last measured MEP, and amplitude ratio (last measured/baseline amplitude). The collected data comprised six numerical values (e.g., MEP amplitude) and eight nominal attributes (e.g., sex).

Intraoperative motor evoked potential monitoring

Intraoperative MEP monitoring was performed using a neurophysiological monitoring system (Neuro Master; Nihon Kohden, Tokyo, Japan). Regarding MEP monitoring, the examiner (e.g., laboratory technician) monitored the waveform and reported to the surgeon if it declined. Disposable nerve conduction study electrodes (Nihon Kohden) were placed in the target muscle on the opposite side of the affected location, including in the abductor pollicis brevis, abductor digiti minimi, anterior tibialis, and abductor hallucis. The muscles to be monitored intraoperatively were determined by the surgeon. When more than one muscle was monitored in patients, we included only the muscle mostly affected by surgery, in consultation with the surgeon, in this analysis.

The motor cortex was stimulated either by transcranial stimulation (strength of stimulation, 50-200 mA; train of stimulation, 5; pulse duration, 0.2-0.5 ms) or by direct cortical stimulation (strength of stimulation, 10-30 mA; train of stimulation, 5; pulse duration, 0.2 ms). Suprathreshold stimulation intensity was used. The baseline MEP was measured before brain tumor excision, followed by MEP waveform monitoring. When the MEP amplitude decrease was greater than 50%, it was reported to surgeons [5,6].

Preprocessing

Our data were imbalanced having fewer cases compared with those without postoperative motor dysfunction. As this might affect the prediction model performance, a synthetic minority oversampling technique (SMOTE) was used [16]. This method, proposed by Chawla et al. [16], was based on the k-nearest neighbors method. In a preliminary study, we used nine different SMOTE percentages (100-900%) and found optimal prediction performance with 800%. Using this as the SMOTE oversampling value, the number of positive samples was increased from eight to 72 instances.

These datasets had a high attribute-to-data ratio. Therefore, only features with significant differences depending on the presence or absence of postoperative motor dysfunction were used for modeling. As the DT algorithm used for evaluating the association between MEP monitoring data and postoperative motor function did not require standardization, the only preprocessing method applied was SMOTE.

Decision tree model development

WEKA version 3.8.6 (Waikato University, Hamilton, New Zealand) was used for data mining. In a preliminary study, several different types of supervised classification methods in the WEKA toolkit were used for predicting postoperative motor dysfunction. Based on previous reports [11,17], we used three basic classification algorithms and an ensemble algorithm. The basic classification algorithm methods were C4.5 (using WEKA’s J48), random forest, and artificial neural network (WEKA’s multi-layer perceptron). The ensemble algorithm used was adaptive boosting (WEKA’s AdaBoostM1) combined with J48. The following parameters were used for the DT model: a confidence factor of 0.16 and a minimum required leaf node sample number of 3.

Classification performance assessment

In this study, the 10-fold cross-validation method was used to develop a prediction model and evaluate classification performance (Figure 1). DT model performance was evaluated by assessing overall accuracy: true-positive + true-negative/(true-positive + true-negative + false-positive + false-negative), sensitivity: true-positive/(true-positive + false-negative), specificity: true-negative/(false-positive + true-negative), kappa (*κ*) coefficient, and area under the receiver operating characteristic (ROC) curve (AUC). The overall accuracy was defined as the ratio of correctly predicted patients. Sensitivity was determined by the percentage of patients wherein postoperative motor dysfunction was correctly predicted. Specificity measured the proportion of patients with favorable postoperative motor function outcomes who were correctly predicted. An ROC curve was constructed by plotting the percentage of true positives versus the percentage of false positives. The optimal classifier should have an AUC approaching 1.0, whereas a value of 0.5 was equivalent to a random guess. *κ* coefficient was used to assess the agreement of the results. Values of *κ* coefficient in the ranges of 0.41-0.60, 0.61-0.80, and 0.81-1.00 were defined as moderate, substantial, and perfect agreement, respectively [18]. Additionally, the algorithm performance was compared using a pairwise comparison between schemes using the standard t-test with the WEKA Experimenter tool.

Additional external validation dataset

Considering external validation of the classification model, we retrospectively collected data from additional patients who underwent brain tumor surgery with MEP monitoring at our hospital between January 2021 and March 2024. Patients with incomplete clinical information were excluded from this study. Ultimately, 28 patients were included in the additional testing dataset (Figure 1).

Statistical analysis

Continuous variables, expressed as means and standard deviations, were compared using the Mann-Whitney U test. Statistical significance was set at p-values <0.05. Fisher’s exact test or the chi-square test was used for comparing categorical variables. We performed binary logistic regression analysis with postoperative motor dysfunction as the outcome variable. Statistically significant variables (p < 0.05) were fitted into the multivariate regression model with a forward-backward stepwise selection method to determine the independent predictors of postoperative paralysis. Bell curve for Excel (Social Survey Research Information Co., Ltd, Shinjuku, Japan) and MATLAB (MathWorks, Natick, MA, USA) were used for statistical analyses.

## Results

Baseline characteristics

**Figure 2:**
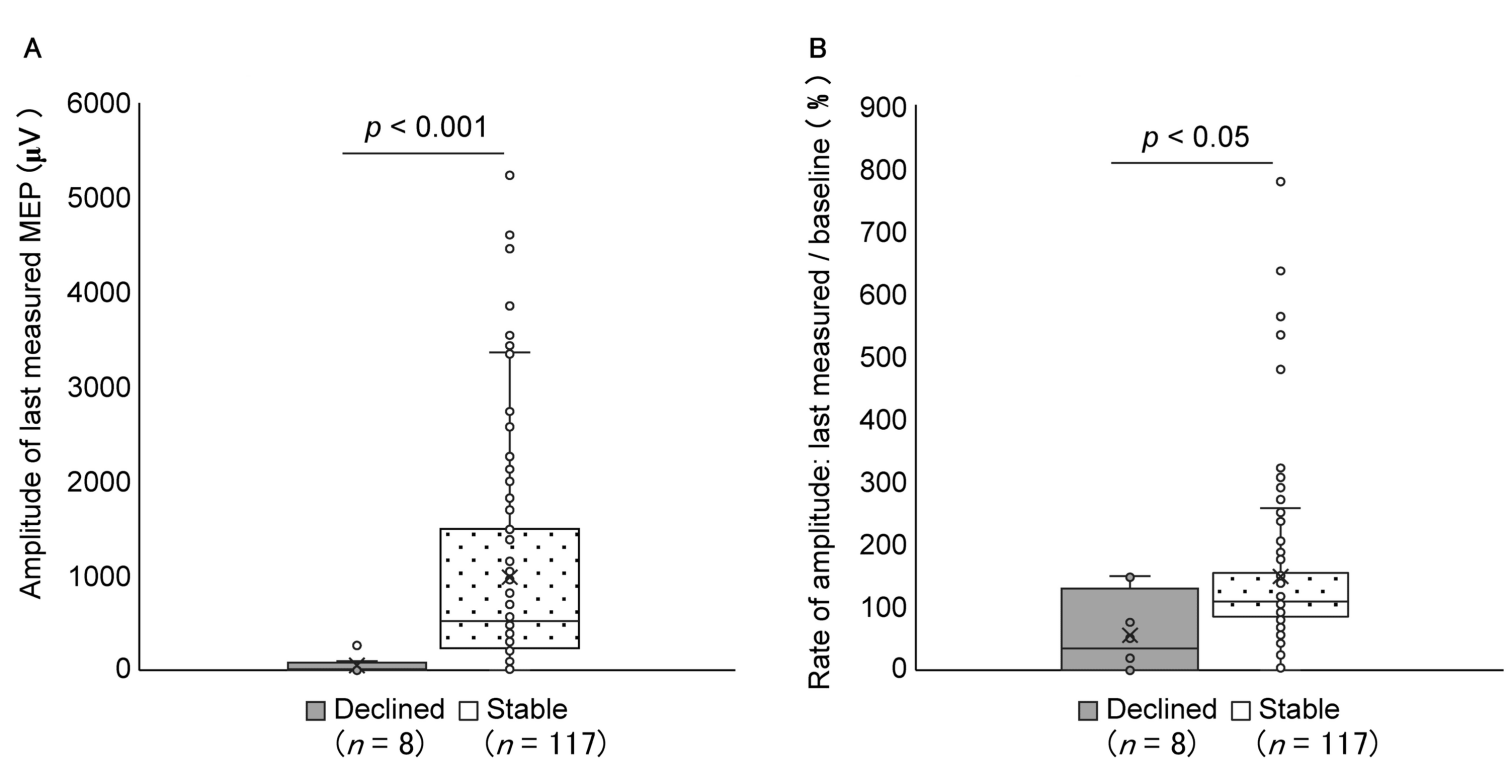
A. The amplitude of the last measured MEP. B. Amplitude ratio: last measured/baseline MEP amplitudes (Mann–Whitney U test). MEP: motor evoked potential

Overall, 129 patients underwent surgery for brain tumor removal with MEP monitoring. Four patients were excluded based on the exclusion criteria; thus, 125 patients were included in this study. This patient group included four pediatric patients aged between six and 15 years. Evaluating the motor function of the muscles monitored by MEP more than two months after surgery revealed that, in eight (6.4%) patients, motor function levels had decreased compared with preoperative levels, whereas 117 (93.6%) patients showed no change in motor function. Patient characteristics are presented in Table 1. The amplitude of the last measured MEP and amplitude ratio were significantly lower in the postoperative motor function decline group (Table 1, Figure 2). Moreover, preoperative motor weakness and changes in intraoperative MEP responses were significantly associated with postoperative motor function.

**Table 1:** Overview of patient characteristics. Continuous variables are expressed as mean (standard deviation). Continuous variables were compared using the Mann–Whitney U test. Fisher’s exact test or the chi-square test was used for comparing categorical variables. Statistical significance was set at p-values <0.05. For statistical analysis methods that do not use test statistics (e.g., Fisher’s exact test), this field was marked as not applicable. N/A: not applicable; MEP: motor evoked potential

Characteristic	Postoperative motor function		Test statistics	P-value
	Stable (n = 117)	Decline (n = 8)		
Age, years	57.6 (18.7)	59.75 (11.1)	U = 448.0	0.844
Sex			χ^2^ = 2.811	0.094
Male	52	6		
Female	65	2		
Body mass index, kg/m^2^	22.7 (3.7)	21.7 (2.3)	U = 404	0.594
Preoperative motor weakness			N/A	0.039
Positive	30	3		
Negative	87	5		
Pathological diagnosis			χ^2^ = 3.942	0.268
Meningioma	54	2		
Glioblastoma	25	4		
Schwannoma	8	0		
Others	30	2		
Side of lesion			χ^2^ = 2.439	0.295
Left	49	3		
Right	51	6		
Others	17	0		
Tumor location			χ^2^ = 5.504	0.239
Frontal lobe	19	1		
Temporal lobe	14	3		
Falx	9	0		
Convexity	18	0		
Others	57	4		
Operative time, min	553.7 (205.9)	583.1 (252.7)	U = 448.5	0.879
Methods of stimulation			N/A	0.147
Transcranial	64	2		
Direct cortical	53	6		
Muscle monitored by MEP			χ^2^ = 0.250	0.732
Abductor pollicis brevis	73	5		
Abductor digiti minimi	24	2		
Tibialis anterior	2	0		
Abductor hallucis	18	1		
Amplitude of baseline MEP, μV	919.8 (1085.9)	394.9 (620.5)	U = 275.0	0.052
Changes in intraoperative MEP response (vs. baseline amplitude)			N/A	0.039
MEP decline >50%	30	5		
MEP decline ≤50%	87	3		
Amplitude of last measured MEP, μV	976.8 (1079.5)	52.7 (92.0)	U = 55.5	<0.001
Amplitude ratio: last measured/baseline, %	149.4 (126.7)	55.7 (63.9)	U = 203.0	0.008

Predictors of postoperative motor dysfunction using multivariable analyses

**Figure 3:**
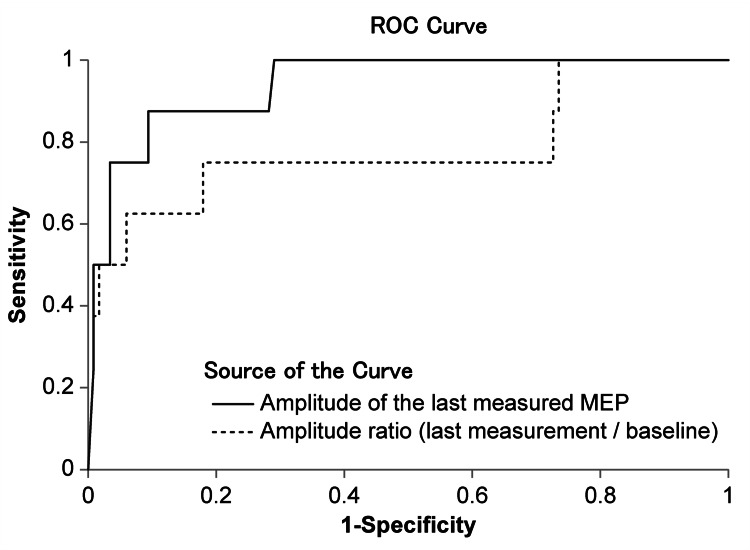
Prediction performance and area under the ROC curves of the amplitude of the last measured MEP and amplitude ratio using internal tests. ROC curves are drawn using these two variables and the AUCs are compared. The two variables are used to calculate a cutoff value to discriminate postoperative motor function. ROC: receiver operating characteristic; MEP: motor evoked potential; AUC: area under the curve

The results of multivariate regression analysis to assess the factors associated with predicting postoperative motor dysfunction are listed in Table 2. The amplitude of the last measured MEP (odds ratio (OR) = 0.9867; 95% confidence interval (CI) = 0.9746-0.9990; p = 0.0344) and the amplitude ratio (OR = 0.9839; 95% CI = 0.9682-0.9998; p = 0.0477) were independent risk factors for the postoperative motor dysfunction. Figure 3 shows the ROC curve for the cutoff value to determine postoperative motor function based on the amplitude of the last measured MEP and amplitude ratio. The ROC curve based on the amplitude of the last measured MEP achieved an AUC of 0.941, a sensitivity of 0.875, and a specificity of 0.906 (p < 0.001). This ROC curve indicated that the appropriate cutoff value was 98.0 μV. The ROC curve for the amplitude ratio showed an AUC of 0.783, sensitivity of 0.750, and specificity of 0.821 (p < 0.014). The ROC curve indicated that the appropriate cutoff value was 76.4%.

**Table 2:** Predictors of postoperative motor dysfunction using binary logistic regression analysis. Model χ_2_: p < 0.01. The multivariate regression model with a forward-backward stepwise selection method was used to determine independent predictors of postoperative paralysis. CI: confidence interval

Characteristic	β coefficient	Odds ratio	95% CI		Wald	P-value
Amplitude of the last measured motor evoked potential	-14.2399	0.9867	0.9746	0.9990	4.4768	0.0344
Rate of amplitude: last measured/baseline	-2.0322	0.9839	0.9682	0.9998	3.9217	0.0477
Preoperative motor weakness	-0.6882	0.2160	0.0292	1.5971	2.2541	0.1333

Prediction performance of the decision tree model

**Figure 4:**
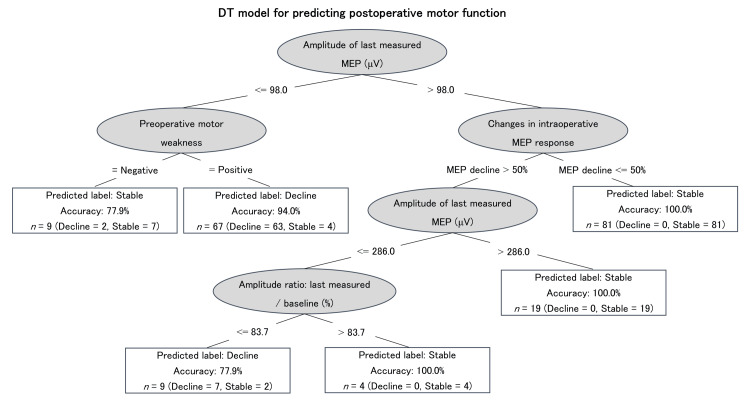
DT model for predicting postoperative motor function and prediction performance using the internal test. Regarding the DT, the elliptical node represents the condition, and the rectangular node represents the predicted outcome, i.e., stable or decline in postoperative motor function. Starting from the root node (i.e., “Amplitude of last measured MEP ≤98 μV”), the corresponding value of the patient is compared with the condition in the root node. If the patient’s last measured MEP amplitude is less than 98 μV, the next node is “Preoperative motor weakness”; alternatively, the algorithm jumps to the “Changes in intraoperative MEP response” node. The patient’s attribute values continue to be compared with other internal nodes of the tree until a rectangular node is reached, at which point the outcome prediction is obtained. The amplitude of the last measured MEP is the root node of the DT. It rates the function of the corticospinal tract and is crucial for predicting postoperative motor function. In patients with the last measured MEP amplitude >98 μV, changes in intraoperative MEP responses are of significance, and the corresponding postoperative motor function will be stable if “Changes in intraoperative MEP response” decline to less than 50%. In patients with the last measured MEP amplitude below 98 μV, preoperative motor weakness plays a significant role, and postoperative motor function would decline if the patient shows preoperative motor weakness. DT: decision tree; MEP: motor evoked potential

Figure 4 shows the DT model of postoperative motor dysfunction. Regarding developing the DT model, variables were selected from the items that showed significant differences or significant associations in patient characteristics. The DT model used the following four variables for postoperative motor function prediction: preoperative motor weakness, changes in intraoperative MEP response, the amplitude of the last measured MEP, and amplitude ratio. As our data were imbalanced, with fewer cases of postoperative motor dysfunction compared with cases of stable motor function, we oversampled using SMOTE to increase the eight cases to 72. Using the internal test, the DT model achieved an overall accuracy of 0.921, sensitivity of 0.917, specificity of 0.921, and AUC of 0.931.

Table 3 presents the prediction performance of the cutoff value from the ROC curve of the amplitude of the last measured MEP and DT model in the 28 external validation datasets. Using the optimal cutoff value of the amplitude of the last measured MEP yielded an overall accuracy of 0.623, sensitivity of 0.500, and specificity of 0.654. However, the DT model achieved an overall accuracy of 0.857, sensitivity of 1.000, and specificity of 0.846. The confusion matrix results are presented in Table 4. The *κ* coefficients, which indicated the degree of agreement between predicted and actual values, were 0.054 (p = 0.662) for the optimal cutoff value of the last measured MEP amplitude and 0.4440 (p = 0.005) for the DT model. Regarding the validation using Fisher’s exact test, the predicted number of fits between the DT model and cutoff values were significantly different (p = 0.010).

**Table 3:** Prediction performance for external validation. ROC: receiver operating characteristic

Prediction method	Accuracy	Sensitivity	Specificity
Cutoff value from the ROC curve (amplitude of the last measured motor evoked potential)	0.623	0.500	0.654
J48 (decision tree)	0.857	1.000	0.846

**Table 4:** Confusion matrix of external validation. ROC: receiver operating characteristic

Cut-off value from the ROC curve		Predicted		J48 (decision tree)		Predicted	
		Decline	Stable			Decline	Stable
Actual	Decline	1	1	Actual	Decline	2	0
	Stable	9	17		Stable	4	22
*κ* = 0.054 (p = 0.662)				*κ* = 0.440 (p = 0.005)			

## Discussion

In this study, a postoperative motor function prediction model was developed using DT analysis of clinical and MEP monitoring data of patients with brain tumors who underwent tumor resection with MEP monitoring. The DT model showed high accuracy for predicting postoperative motor function after brain tumor resection when applied to an additional independent dataset. In this DT model, all elements used to make forecasts and their selection criteria are explicitly described. This high explanatory power is an advantage of this model. The results indicated that this DT-based model was more useful for predicting postoperative motor function than using the traditional cutoff MEP amplitude values from ROC curves.

Among the ML algorithms, the DT model applied in this study is easy to read, providing interpretable and logical rules. Furthermore, the DT model results indicated important classification attributes. These characteristics of DT analysis make it suitable for the analysis of MEPs, which are affected by various factors. The conventional prediction of postoperative paralysis based on MEP amplitude values and amplitude ratios is difficult because the multiple factors involve complicated evaluation. In contrast, DTs use multiple factors to build a predictive model and improve accuracy, demonstrating the clinical usefulness of the DT model.

Furthermore, a unique feature of this study was the use of the SMOTE oversampling technique. This technique has previously been used to improve the accuracy of a prediction model for diabetes development based on cardiopulmonary function recordings [19]. In our preliminary study, the classification performance of the DT model was also improved using SMOTE, increasing the eight identified cases to 72 postoperative paralysis cases. When standardization and SMOTE were applied for preprocessing, AdaBoostM1 demonstrated the highest accuracy, sensitivity, and *κ* coefficient (Table 5). However, the DT model showed no significant difference in classification performance compared with other complex algorithms, such as AdaBoostM1 (Table 6).

**Table 5:** Classification performance by preprocessing conditions and algorithms. AUC: area under the curve; RF: random forest; MLP: multi-layer perceptron; AdaBoostM1: adaptive boosting

Preprocess	Algorithm	Accuracy	Sensitivity	Specificity	*κ* coefficient	AUC
Standardization	J48	0.915	0.178	0.138	0.195	0.637
	RF	0.918	0.134	0.138	0.264	0.877
	MLP	0.920	0.065	0.225	0.204	0.936
	AdaBoostM1	0.909	0.083	0.100	0.153	0.866
	Mean	0.915	0.115	0.150	0.204	0.829
Standardization + synthetic minority oversampling technique	J48	0.913	0.876	0.918	0.819	0.927
	RF	0.916	0.857	0.927	0.824	0.973
	MLP	0.906	0.884	0.916	0.807	0.970
	AdaBoostM1	0.921	0.898	0.916	0.833	0.964
	Mean	0.914	0.879	0.919	0.821	0.959

**Table 6:** Comparison of prediction performance for J48 and AdaboostM1. The algorithm performance was compared using a pairwise comparison between schemes using the standard t-test with the WEKA Experimenter tool. This analysis adopted a significance level of 0.05. AUC: area under the curve; AdaBoostM1: adaptive boosting

Algorithm	Accuracy	Significant	Sensitivity	Significant	Specificity	Significant	*κ *coefficient	Significant	AUC	Significant
J48	0.906	No	0.885	No	0.886	No	0.801	No	0.928	No
Adaboost M1	0.920		0.908		0.891		0.830		0.972	

The resulting DT model was superior to the ROC of the last measured MEP amplitude in terms of sensitivity and specificity using the internal test. The first branch of the DT model was the last measured MEP amplitude and used a value of 98 μV for this variable as a criterion. The results of the DT model, which showed more cases of postoperative motor dysfunction when the last measured MEP amplitude was below 98 μV, supported the relationship between MEP amplitude and postoperative motor function. An amplitude of 98 μV in the last measured MEP was also the cutoff value calculated by the ROC curve. Furthermore, several reports have shown that the MEP amplitude criterion for predicting permanent motor dysfunction is less than 100 µV [20-22]. Thus, the findings of these reports and the criterion for the first branch of DT in this study were similar. Many studies have used an amplitude decrease of greater than 50% of the baseline waveform as a warning criterion [9]. In this study, the amplitude ratio formed the fourth branch of the DT, suggesting that it was not as important as the amplitude of the last measured MEP for predicting paralysis. This may be due to the divergence of baseline amplitude and preoperative motor functions.

According to the external validation results (Table 3), the DT model showed better classification performance compared with that based on the ROC curve cutoff value. Based on the confusion matrix, only the *κ *coefficient of the DT model was significant (Table 4). Fisher’s exact test showed that the predicted number of fits for the DT model and cutoff values were significantly different. These results indicated that applying the DT model could have the potential to reduce false positives, which are often problematic for MEP monitoring. These results can be attributed to ML algorithms, such as DT models, which use multiple factors to make predictions. In other words, for predicting events involving multiple variables, such as MEP monitoring, the results suggest that ML is effective. Another feature of DT modeling is its ability to accommodate both continuous and categorical variables. Due to these advantages, DT analysis is widely used in biomedical sciences [23,24]. For example, DT models have been used in neurosurgery to predict long-term outcomes after poor-grade aneurysmal subarachnoid hemorrhage [13] and identify prognostic factors for survival in patients with recurrent glioblastoma [15]. The DT model is a visual representation of the decision-making process and can be easily applied and interpreted. Considering these features, a DT model using MEP monitoring data, such as the one in this study, may aid in clinical decisions, such as determining the extent of intraoperative resection of tumors and choice of postoperative therapy, and may help develop guidelines for evaluating MEP monitoring data. However, the clinical integration of the DT model among other facilities is currently difficult due to challenges in standardization and generalizability. It may be possible for each institution to develop its own DT model, validate it, and implement it in clinical practice.

This study has some limitations. First, this was a retrospective study and, despite its promising results, additional prospective studies are needed. Second, this study was conducted at a single institution; thus, further multicenter studies are needed to generalize the predictive model using DTs. Third, although the motor function assessments in this study were performed more than two months after surgery, the assessment timing was not consistent, preventing the exclusion of rehabilitation effects. Fourth, the external validation sample size was small, with only two paralytic cases. Therefore, the accuracy and sensitivity estimates in our external validation may be unreliable, hindering the generalization of the DT model. Fifth, postoperative data (e.g., laboratory data) were not used for model creation. In addition, other confounding factors that might have affected prediction accuracy included the amount of intraoperative blood loss, intraoperative blood pressure, amount of intravenous anesthesia used, consciousness status before and after surgery, performance differences between monitoring devices, and differences in postoperative therapy. In the future, creating a more accurate model by using such data might be possible. Finally, the participants’ MEP monitoring data were produced by a mixture of transcranial and cortical stimulation, with different stimulation parameters, which may have affected the results. Further studies are needed to validate these findings, ideally using a larger cohort with a unified stimulation method and incorporating postoperative examination data as an additional feature in model reconstruction. Moreover, prospective studies may further clarify the utility of the DT model by comparing its paralysis prediction performance with that of the existing models across multiple consistent time points.

## Conclusions

We developed a DT model for predicting paralysis that used MEP monitoring data of patients who underwent brain tumor surgery. This model demonstrated high accuracy for predicting postoperative motor function using both internal and external validation tests. The DT model is simple to use and can be used to predict postoperative motor function in patients undergoing brain tumor resection using intraoperative MEP monitoring. Although prospective and multicenter studies are needed for generalization purposes, this DT model may assist in the planning of treatment strategies by predicting postoperative motor function in various patients.
